# Generalized Radar Range Equation Applied to the Whole Field Region

**DOI:** 10.3390/s22124608

**Published:** 2022-06-18

**Authors:** Luyin Xiao, Yongjun Xie, Shida Gao, Junbao Li, Peiyu Wu

**Affiliations:** 1 School of Electronic and Information Engineering, Beihang University, Beijing 100191, China; xiaoluyin0626@163.com (L.X.); yjxie@buaa.edu.cn (Y.X.); sy2002106@buaa.edu.cn (S.G.); 2 Beijing Key Laboratory of Microwave Sensing and Security Applications, Beihang University, Beijing 100191, China; 3 Shenzhen Institute of Beihang University, Shenzhen 518000, China; 4 School of Electronics and Information Engineering, Harbin Institute of Technology, Harbin 150080, China; junbaolihit@126.com

**Keywords:** THz radar system, generalized radar range equation, near-field RCS, near-field radar theory

## Abstract

Most terahertz (THz) radar systems can only work in the near-field region, because the THz source power is limited and the size of the target scattered near field is up to tens of kilometers. Such conditions will result in the conventional radar range equation being unsuitable. Therefore, the near-field radar cross section (RCS) formula is given according to the numerical simulation on different targets. By modifying the parameters in the near field, including the gain of radar antennas and the RCS of targets, the generalized radar range equation is proposed. The THz radar working efficiency in the whole range and the simulation of the near-field RCS simulation model were employed to validate its effectiveness. Through comparison with the radar range equation, it can be concluded that the calculation results of the proposed equation are smaller in the near field, and the outcomes in the far field are identical. The proposed generalized radar range equation can be applied to the whole radiation area including the near field and the far field. Furthermore, more complicated real targets are calculated according to the generalized radar range equation and it can be extended from the submillimeter wave band to a much wider band range. Finally, the near-field radar theory is established, which shows its potential application to the radar cross section estimation in the extremely high frequency and fine design of THz radar systems.

## 1. Introduction

**Figure 1:**
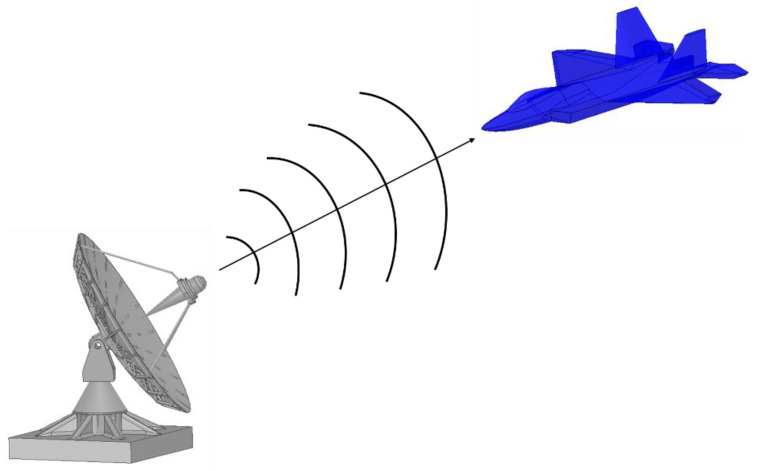
Monostatic THz radar system detecting a target in the near field.

Submillimeter (terahertz) waves, with the frequency range from 0.1 to 10 THz, have features of wide frequency bandwidth, high data rate, and high spectral resolution [1,2]. Because of these unique characteristics, terahertz waves have been applied to various domains including wireless communication, industry, and imaging [3,4,5]. THz radar, as essential research in target detection, has been receiving increasing attention among these applications. Compared with the microwave radar, THz radar systems have a higher carrier frequency, larger absolute bandwidth, higher data rate, and smaller dimension [6,7,8]. However, because of the huge difference between the size of targets and the wavelength in the submillimeter wave band, the scattered near-field region of the target is up to tens of kilometers in size. Therefore, the electromagnetic scattering should not be approximated by the plane wave when the THz radar system detects a target, as shown in Figure 1. In addition, power generation is a fundamental problem for THz radar systems [9,10]. The source of the THz radar emitters is in the watt level, which means most of the THz radar systems can only work in the near-field region. The radar range equation used in the far field is not applicable.

There have been several studies on THz radar systems [11,12]. Cooper developed a 580 GHz radar by employing frequency-modulated continuous wave (FMCW) radar technique and presented a 675 GHz radar with 28.8 GHz bandwidth, which can detect objects concealed underneath clothing [13,14,15]. The mechanical translation method of the transceiver is used to provide a THz radar with refocusing capabilities, which leads to very good imaging quality at the range from 12.5 m to 37.5 m [16]. Murano and Watanabe developed a short-range THz radar with a leaky-wave antenna, which has beam steering capability; it can be integrated with a detector and a solid-state source and applied to small devices [17]. Most of THz radars are applied in the near field, but there is no relevant equation or theory to analyze them. Therefore, it is essential to propose a near-field radar theory suitable for the entire range, which has a good effect on the precise design of THz radars.

As for the radar range equation, it can be applied to estimate the radar cross section (RCS), but the RCS is estimated in the far field [18]. The unique RCS characteristic is calculated to predict a target state based on the radar equation for 26 GHz and 79 GHz short range vehicular radars. However, this equation only changes the range-bin ΔR; the concept of other parameters including gain and RCS are not suitable for this short range [19]. Other refinement of the radar range equation is due to the slow decay effect in radiation for a wide-spectrum short-pulse radar [20] or moving target imaging for synthetic aperture radar (SAR) [21,22]. Nevertheless, none of these modifications of the radar range equation are well applied to the near-field zone. Thus, a novel radar range equation needs to be derived.

This article is organized as follows. In Section 1, we derive the generalized radar range equation in detail. Section 3 analyzes much more complicated targets for the validation of the generalized radar range equation. The near-field radar theory is summarized in Section 4. Finally, Section 5 provides the conclusion of this paper.

## 2. Generalized Radar Range Equation

For traditional monostatic radar systems, the radar range equation is given as [23]

(1) $$\eta =\frac{P_{r}}{P_{t}}=\frac{G_{t}G_{r}\lambda^{2}\sigma }{{\left(4\pi \right)}^{3}R^{4}}$$

where *P_r_* is the received power; *P_t_* is the peak transmitted power; η is the ratio of these; *G_t_* and *G_r_* are the gain of the transmitting and receiving antenna, respectively; λ is the wavelength; σ is the RCS of the target; and *R* is the range from the radar to the target. Assuming the RCS (σ dBsm) of a target is 30 dBm^2^, the gain of both the transmitting antenna and the receiving antenna is 45 dB and the operating frequency is 1 THz. According to radar Equation (1), if the range is R=10λ, R=100λ, and R=1000λ, then the ratio of *P_r_* to *P_t_* will be η=96.6 dB, η=56.6 dB, and η=16.6 dB, respectively. It should be noticed that η is far more than 0 dB in the near-field region. Like the gain of antennas [24], it is unreasonable to assume the RCS value of a target is constant in such a near range from 10λ to 1000λ. Thus, an alternative radar range equation applied to the THz radar system needs to be derived.

According to the previous research, we have defined the near-field gain and its expression has been derived in [24]. The near-field gain $G_{N}$ and RCS defined in the near-field region $\sigma_{N}(R)$ are substituted into the radar range equation as

(2) $$P_{r}=\frac{P_{t}G_{NT}(R_{0})G_{NR}(R_{0})\lambda^{2}\sigma_{N}(R)}{{\left(4\pi \right)}^{3}R^{4}}$$

(3) $$G_{N}=\frac{4\pi }{\lambda^{2}}\cdot \eta_{NA}\cdot A_{f}$$

(4) $$\eta_{NA}=\frac{\left|I\left(m,n\right)+c_{n}I\left(m,0\right)+c_{m}I\left(0,n\right)+c_{m}c_{n}I\left(0,0\right)\right|}{2\sqrt{\left(\frac{{c_{m}}^{2}}{2}+\frac{c_{m}}{m+1}+\frac{1}{4m+2}\right)\left(\frac{{c_{n}}^{2}}{2}+\frac{c_{n}}{n+1}+\frac{1}{4n+2}\right)}}$$

where $G_{NT}(R_{0})$ and $G_{NR}(R_{0})$ are the near-field gain of the transmitting antenna and the receiving antenna, respectively; $R_{0}$ is the distance between these two antennas; *A_f_* represents the antenna effective area; and coefficient $\eta_{NA}$ is the aperture utilization efficiency of the near-field antenna. *I*(*m*,*n*) within (4) is $I(m,n)=n!m!\sum_{j=0}^{\infty }\left[{\left(-1\right)}^{j}{\left(\mu /2\right)}^{2j}\right]/\left[(j+m+1)!(j+n+1)!\right]$ and $\mu =kr_{0}{r_{0}}^{\prime }/R_{0}$. *m*, *n*, *c_m_*, and *c_n_* are parameters related to the aperture distribution of the radar antennas; $r_{0}$ and ${r_{0}}^{\prime }$ are radius of the transmitting and receiving aperture antenna.

The RCS of different metal targets including sphere, plate, cylinder, and conical frustum in the near field was obtained by numerical simulation, using the near-Greco algorithm.

**Figure 2:**
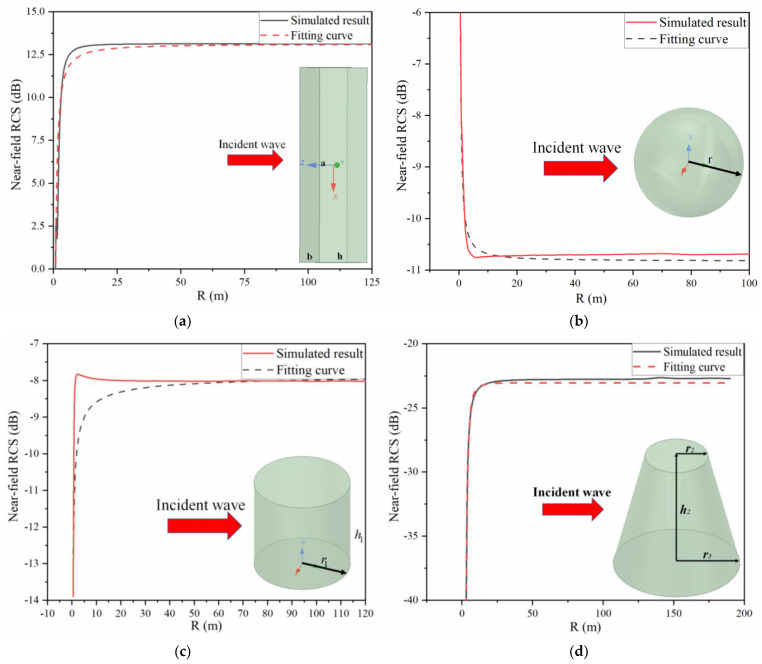
Numerical simulated results and fitting curves of the near-field radar cross section for different targets. (**a**) Plate; (**b**) sphere; (**c**) cylinder; (**d**) conical frustum.

Figure 2 shows the numerical simulation results and corresponding fitting curves of the near-field radar cross section for these metal targets. The inserted map in Figure 2 shows the model of these metal targets. The radius of the sphere *r* is 0.2 m; the size of the plate a×b×h is 0.2 m×0.2 m×0.05 m; whereas for the cylinder, *r_1_* is 0.1 m and its height is 0.1 m, and the dimension of the conical frustum is 0.05 m×0.1 m×0.2 m. It can be found that the direction of all of the incidence waves is perpendicular to the vertical section. The discrepancy between the numerical simulation and fitting curves for these targets is less than 0.5 dB.

In conclusion, the RCS formulas of these targets in the near field can be given as

(5) $$\sigma_{NP}=10\mathrm{lg}\left(\frac{4\pi a^{2}b^{2}}{\lambda^{2}}\right)+k_{NP}\cdot {\left(Rab\lambda \right)}^{-1.17}+\Delta_{P}$$

(6) $$\sigma_{NS}=10\mathrm{lg}\left(\pi r^{2}\right)+k_{NS}{\left(\frac{R}{r}\right)}^{-1.15}\lambda^{-0.37}+\Delta_{S}$$

(7) $$\sigma_{NC}=10\mathrm{lg}\left(\frac{2\pi r_{1}{h_{1}}^{2}}{\lambda }\right)+k_{NC}R^{-0.61}{\left(\frac{h_{1}}{r_{1}}\right)}^{1.5}+\Delta_{C}$$

(8) $$\sigma_{NF}=-23.04+k_{NF}\cdot R^{-2.93}+\Delta_{F}$$

where $\sigma_{NP}$, $\sigma_{NS}$, $\sigma_{NC}$, and $\sigma_{NF}$ are the near-field RCS of the plate, sphere, cylinder, and conical frustum, respectively; coefficient $k_{NP}=-4.4\times 10^{-3}$; $k_{NS}=4.05$; $k_{NC}=-3.23$; and $k_{NF}=-425.02$. $\Delta_{P}$, $\Delta_{S}$, $\Delta_{C}$, and $\Delta_{F}$ are the correction factors, which may be different with the change in the frequency and the size of targets. However, the discrepancies are so tiny among the same kind of target that they could be neglected.

Thus, the near-field RCS can be explicated as the far-field RCS with the same direction and a function related with the detection range *R* and the target dimension *D*, given as

(9) $$\sigma_{N}(R,D)=\sigma +k*f(R,D)*\lambda^{n}+\Delta$$

where coefficient *k* and *n* are constants, f(R,D) is the function related with range and targets’ dimension, and Δ is the correction factor.

For monostatic radar systems, transmitting antennas and receiving antennas are the same, so both the gain $G_{NT}(R_{0})$ and $G_{NR}(R_{0})$ can be replaced by *G_N_*(*R*_0_). Replacing $\sigma_{N}(R)$ in Equation (2) by RCS Formulas (5)–(7), the generalized radar range equation for the plate, sphere, and cylinder can be expressed as

(10) $$P_{r}\left(\mathrm{dB}\right)=P_{t}(\mathrm{dB})+20\mathrm{lg}G_{N}(R_{0})+20\mathrm{lg}\left(\frac{ab}{R^{2}}\right)+k_{NP}{\left(Rab\lambda \right)}^{-1.17}+\Delta_{P}-21.98$$

(11) $$P_{r}\left(\mathrm{dB}\right)=P_{t}(\mathrm{dB})+20\mathrm{lg}G_{N}(R_{0})+20\mathrm{lg}\left(\frac{r\lambda }{R^{2}}\right)+k_{NS}{\left(\frac{R}{r}\right)}^{-1.15}\lambda^{-0.37}+\Delta_{S}-28.01$$

(12) $$P_{r}\left(\mathrm{dB}\right)=P_{t}(\mathrm{dB})+20\mathrm{lg}G_{N}(R_{0})+10\mathrm{lg}\left(\frac{r_{1}{h_{1}}^{2}\lambda }{R^{4}}\right)+k_{NC}R^{-0.61}{\left(\frac{h_{1}}{r_{1}}\right)}^{1.5}+\Delta_{C}-25.00$$

From Equations (10)–(12), we can find that the parameters including near-field gain and near-field RCS are related to the distance from the radar to the target, except for the wavelength and the dimension of targets, which is totally different from radar range Equation (1) defined in the far field. In conclusion, for more complicated targets, the general form of the generalized radar range equation can be represented as

(13) $$P_{r}\left(\mathrm{dB}\right)=P_{t}(\mathrm{dB})+20\mathrm{lg}G_{N}(R_{0})+10\mathrm{lg}\sigma_{N}(R)+20\mathrm{lg}\left(\frac{\lambda }{R^{2}}\right)-32.98$$

where the near-field $G_{N}(R_{0})$ and near-field RCS $\sigma_{N}(R)$ are given in (3) and (9), respectively; the constant term 32.98 is the calculation result of $10\mathrm{lg}\left({\left(4\pi \right)}^{3}\right)$.

## 3. Verification and Discussion

To validate the performance and correctness of the proposed generalized radar range Equation (13), the efficiency of the THz radar, the unity of the generalized radar range equation in the whole range, and the calculation for complicated targets are employed. Assuming even aperture distribution of transmitting and receiving antennas in this chapter, the aperture utilization efficiency can be the highest.

### 3.1. THz Radar Working Efficiency

**Figure 3:**
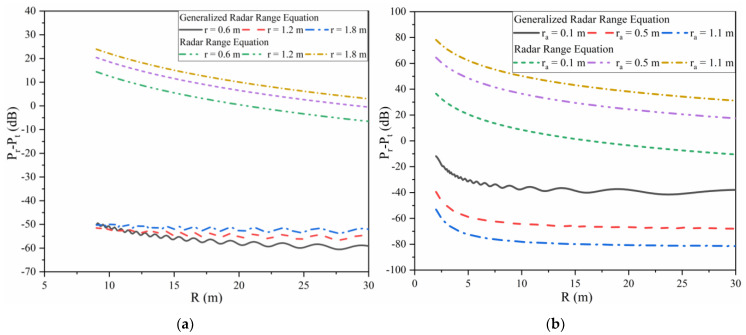
(**a**) THz radar detects different sizes of metal spheres. (**b**) Different THz radars detect the same metal target.

The value of $\left(P_{r}-P_{t}\right)$ dB is defined as THz radar working efficiency in the near field, which should be less than 0 dB. Figure 3a shows a THz radar detecting different sizes of metal spheres in the near field. The wavelength of the THz radar is 0.2 mm and the radius of the antenna aperture is 0.3 m.

As shown in Figure 3a, the value of $\left(P_{r}-P_{t}\right)\;\mathrm{dB}$ calculated by the radar range equation is larger than zero and the maximum value is over 30 dB, which is unreasonable. This is because the electromagnetic wave received by the receiving antenna hugely exceeds the power emitted by the transmitting antenna. However, the value of $\left(P_{r}-P_{t}\right)\;\mathrm{dB}$ calculated by Equation (13) is less than 0 dB in the near-field region. Furthermore, with the expansion of the target’s volume, the THz radar working efficiency increases. This rule proves the correctness of the generalized radar range equation applicable to the near-field region, because it is easier for a radar to detect larger objects.

Figure 3b shows THz radars with different sizes of the transmitting and receiving antennas’ aperture detecting the same metal target in the near field. The size of the metal target is 1.7 m and the wavelength of the THz radar is 0.1 mm. Here, *r_a_* is the radius of the antenna’s aperture for monostatic THz radar systems.

Similar to Figure 3a, the results in Figure 3b calculated by Equation (1) are greater than 0 dB in the near field. Additionally, the THz radar working efficiency may be higher if the antenna chooses a smaller aperture. However, it may need beam scanning to detect a larger target. All of these conclusions indicate the effectiveness of the generalized radar range equation.

### 3.2. Unity of the Generalized Radar Range Equation

**Figure 4:**
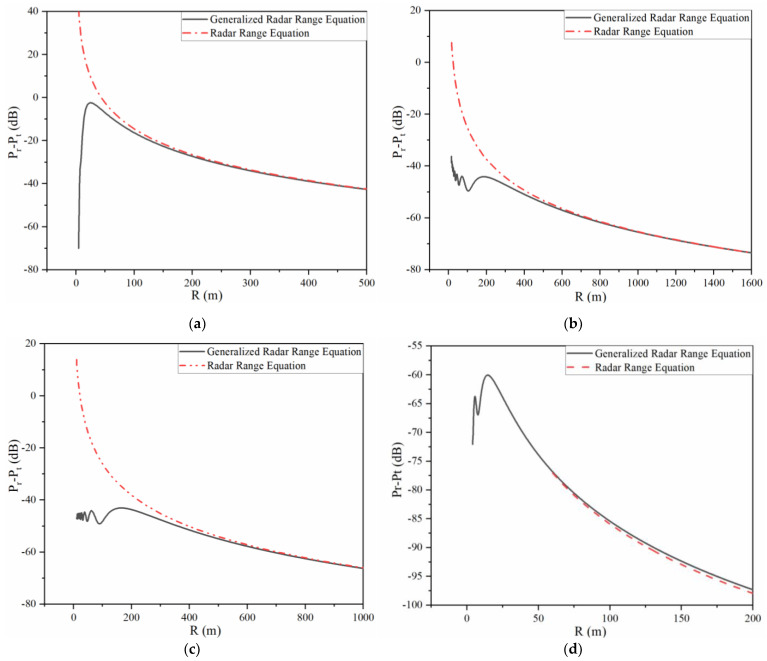
Relationship between $\left(P_{r}-P_{t}\right)\;\mathrm{dB}$ and R in the whole range. (**a**) Plate, (**b**) sphere, (**c**) cylinder, (**d**) conical frustum.

The unity of the proposed generalized radar range equation is demonstrated through the fitness with the radar range equation in the far field, assuming the maximum value of the RCS for normal incidence can be obtained in (1) [25]. Figure 4 shows the relationship between the $\left(P_{r}-P_{t}\right)\;\mathrm{dB}$ and range R in the whole range; the results of $\left(P_{r}-P_{t}\right)\;\mathrm{dB}$ are obtained by Equation (1) and generalized radar range Equation (13), respectively.

In the far-field zone, the RCS of a plate becomes [26]

(14) $$\sigma_{FP}=\frac{4\pi a^{2}b^{2}}{\lambda^{2}}$$

In this experiment, the size of the plate is a⋅b=0.3 m×0.2 m; for monostatic THz radar systems, the wavelength is 1 mm and the radius of two identical aperture antennas is 0.1 m. As shown in Figure 4a, the value of the purposed radar equation is smaller than that of Equation (1) and augments in the near field with the increase in R. When the range R approaches infinity, these two curves tend to be coincident. The value of $\left(P_{r}-P_{t}\right)\;\mathrm{dB}$ in the far-field zone calculated by Equation (13) is a variable only proportional to $R^{4}$, which has the same form as Equation (1).

In the far-field region, the RCS of a sphere is independent of the wavelength if operating at a high frequency. Therefore, the RCS of a sphere can be given as [27]

(15) $$\sigma_{FS}=\pi r^{2}$$

The aperture size is 0.24 m and the operating frequency is 600 GHz for monostatic THz radar systems, and the radius of the detected sphere target is 3 m. It can be observed in Figure 4b that the traditional radar range equation is irrational in the near field owing to the positive value. The curve of Equation (13) in the near field has irregular vibration and comes close to the dash-dotted line in the far-field region. With the increase in the range R, the THz radar working efficiency gradually declines.

Similarly, the RCS of a cylinder in the far field is given by the following formula [28]:

(16) $$\sigma_{FC}=\frac{2\pi r_{1}{h_{1}}^{2}}{\lambda }$$

In this model, the wavelength is 0.1 mm and the aperture radius is 0.1 m. The radius of this target is 0.05 and its height is 0.1 m. It can be discovered from Figure 4c that the curve of Equation (13) fluctuates with the value of lower than 0 dB in the near field. Such fluctuation in both (b) and (c) reveals that many elements affect the value of $\left(P_{r}-P_{t}\right)\mathrm{dB}$. The solid line (Equation (11)) and the double-dotted line (Equation (1)) are falling in the far field and can gradually overlap.

The monostatic RCS of this conical frustum is −23.70 dB through the numerical simulation in the far field, which is approximate to the constant term of Expression (8). The calculation result for this model is displayed in Figure 4d. It can be observed that the THz radar efficiency sways in the Fresnel region and then declines with the increasing distance. Further, the figure of Equation (13) is nearly coincident with the radar range equation in the far-field region with a maximum discrepancy of about 1 dB.

In conclusion, the generalized radar range equation can be used in the Fresnel zone. Meanwhile, the purposed radar equation in the far-field region is converted to the traditional radar range Equation (1). Therefore, the proposed generalized radar range equation can be applied to the whole radiation area.

### 3.3. Comparison with Near-Field RCS Simulation Model

**Figure 5:**
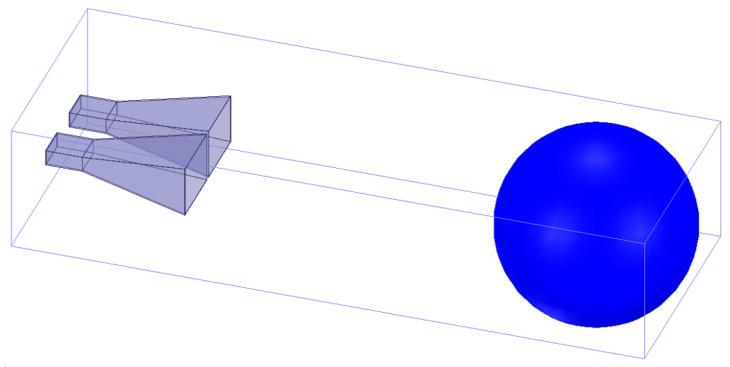
Near-field RCS simulation model.

To further demonstrate the effectiveness of the proposed equation, the comparison with the near-field RCS simulation model is adopted. The model shown in Figure 5 is composed of the transmitting and receiving antenna with 10 GHz and detected targets. The full-wave simulation software is used to analyze this model.

**Figure 6:**
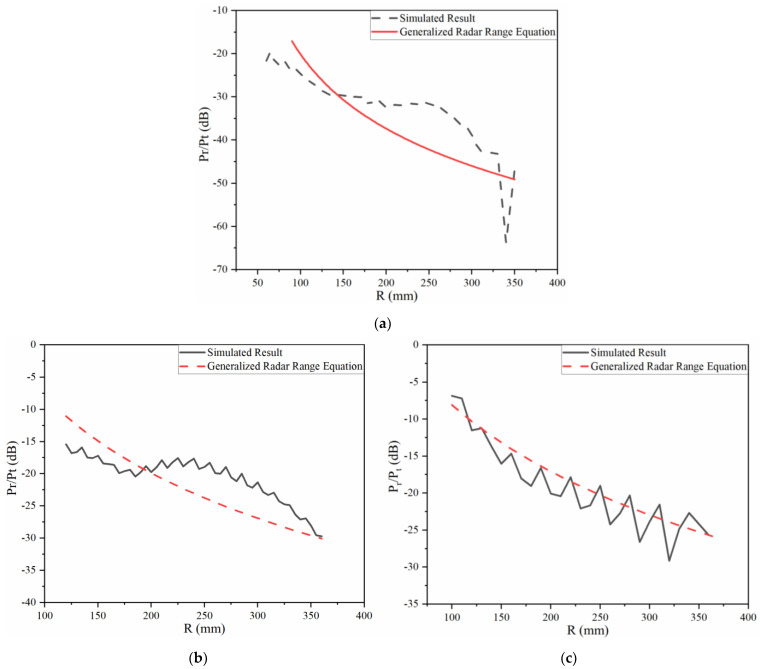
Results of $\left(P_{r}/P_{t}\right)\;\mathrm{dB}$ obtained by the full-wave simulation and the generalized radar range equation. (**a**) Metal sphere, (**b**) metal plate, (**c**) metal cylinder.

Figure 6a shows the results of $\left(P_{r}/P_{t}\right)\;\mathrm{dB}$ when the target is a metal sphere in this model. It can be observed that the tendency of $\left(P_{r}/P_{t}\right)\;\mathrm{dB}$ through the full-wave simulation analysis and generalized radar range equation hold the same form. The maximum error between the simulated analysis and calculation result is less than 4 dB, which indicates the accuracy of the purposed Equation (13). The error between different results is firstly due to the unknown aperture field distribution. In addition, the transmitting and receiving antenna cannot be placed in the same position because of the restriction of this model. Thus, the scattered electric field of the target and the incident electric field from the transmitting antenna cannot remain in the same direction and ensures that the incident wave is vertical incidence. The value of the scattered electric field appears to be deviation.

Figure 6b is the detected object changed to the metal plate. It can be found that the curves of numerical analysis and the purposed equation are coincidental with the deviation of 3 dB. Except for the above-mentioned error analysis, the thickness of a metal plate may affect the simulated analysis, which is not considered in (5). The distance range in Figure 6b may be close to the reactive near field area, which can cause the oscillation of curve. Although there is no excitation in the receiving antenna, the mutual coupling still exists.

The simulated results obtained from electromagnetic simulation software and theoretical calculation results of the same cylinder target are displayed in Figure 6c. Both of the curves hold the same tendency and the biggest discrepancy between the simulated and theoretical results is about 4 dB. The effectiveness of the proposed Equation (12) was affirmed through this model. However, the electromagnetic simulation software itself also has simulation errors, which will have a bad effect on the accuracy of the simulated results in this model. The limitations of the near-field RCS simulation model can also explain this discrepancy.

### 3.4. Calculation for Complicated Targets

Some real complicated targets are calculated to prove the practicality and universality of (9) and (13) in engineering. It was proved in the previous section that the efficiency in the Fresnel region according to the radar range equation is poor, under only the far-field results of $\left(P_{r}-P_{t}\right)\;\mathrm{dB}$, and is calculated by Equation (1).

**Figure 7:**
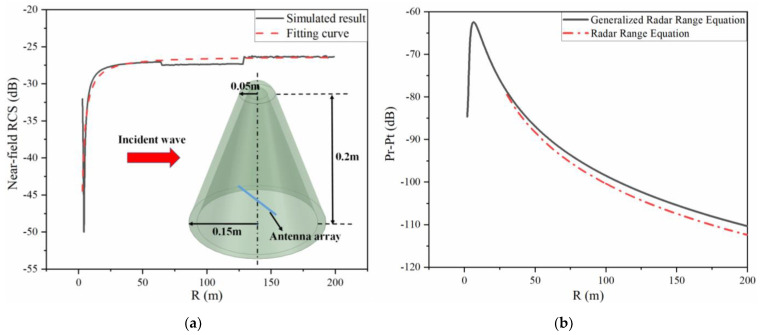
(**a**) Near-field RCS results for high-order spherical cone radome. (**b**) Calculation result of Equation (13).

Radome is one of the most general targets to be detected by radar systems, which is a cover to protect antennas from the physical environmental disturbance and needs to meet the aerodynamic requirements [29]. Figure 7a is the near-field RCS outcome of the high-order spherical cone radome and the inserted map shows its dimension. The near-field RCS fitting formula at 9.6 GHz with normal incidence can be derived as

(17) $$\sigma_{NB}=-26.30+k_{NB}\cdot R^{-1.16}+\Delta_{R}$$

where coefficient $k_{NB}=-64.79$ and $\Delta_{R}$ are the correction factors. The monostatic RCS of this model is −28.50 by numerical simulation with the same incident angle.

The calculation result according to the generalized radar range equation is shown in Figure 7b. It can be observed that the radar working efficiency in the Fresnel zone is improved with the increase in the detection range, which is totally opposite to the concept of radar detection. The radar working efficiency in the far field decreases with the change in the range *R* and approximately equals to the results calculated by Equation (1), where the error is less than 1.5 dB.

**Figure 8:**
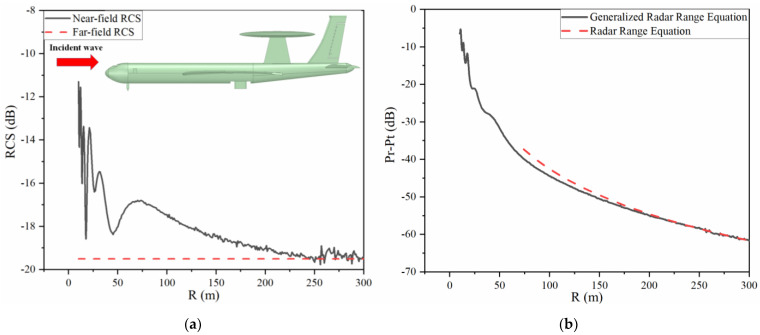
(**a**) RCS results for the early warning aircraft in the whole region. (**b**) Calculation results of Equation (13).

Figure 8a shows the whole region RCS results of the early warning aircraft. The value of far-field RCS is −19.51 dB through numerical simulation and the near-field RCS approaches it in the far field. Figure 8b displays the $\left(P_{r}-P_{t}\right)\;\mathrm{dB}$ results computed by the generalized radar range equation in the whole range and (1) in the far field. It can be found that fluctuation of the curve occurs in the Fresnel zone and the overall trend gradually drops down and fits the dotted line. The discrepancy between these two lines in the far-field region is about 0.1 dB.

In conclusion, the generalized radar range equation can be applied to analyze more complicated real targets in its working area and its rationality is affirmed by the unity with the radar range equation in the far-field region. Furthermore, the purposed equation can be extended to the millimeter wave band for a radar system.

## 4. Near-Field Radar Theory

Through the demonstration of this article, the generalized radar equation theory can be concluded. The proposed theory is composed of the near-field gain, the near-field RCS, and the generalized radar range equation.

**Figure 9:**
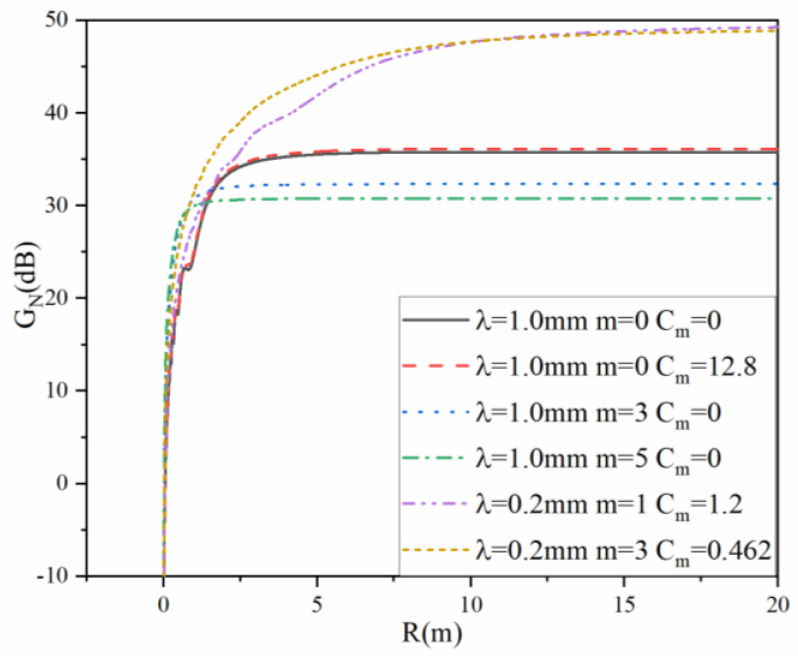
Near-field gain of transmitting and receiving antennas for the radar system with different aperture field distributions.

The near-field gain for the radar system is defined in [24], which is related to the distance, diameter, aperture illumination, and wavelength in the Fresnel zone. In the far-field region, the near-field gain is a fixed value without distance and other parameters, which is consistent with the far-field gain definition. Figure 9 displays the near-field gain results of aperture antennas for the radar system [24].

The near field RCS Expression (9) is composed of the RCS with the same direction defined in the far field, the function related with the range R, target dimension, frequency, and the error due to the characteristic in the near-field region. When the range approaches the far-field region, the function is close to the RCS measured in the far-field region. Similar to the near-field gain, the near-field RCS shows its unity in these phenomena.

The generalized radar range equation is the widespread use of the near-field gain expression. Substituting the gain and the RCS of the radar range equation with the above near-field gain and near-field RCS, it can be derived. The calculation result $\left(P_{r}-P_{t}\right)\;\mathrm{dB}$ in the near field is below 0 dB and smaller than that calculated by Equation (1). Moreover, the generalized radar range equation can be simplified into Equation (1) in the far field.

## 5. Conclusions

In this paper, the generalized radar range equation is proposed, which combines numerical simulation with parameters’ modification. The proposed equation is the wide promotion of the near-field gain expression. It is applicable to the whole radiation area and solves the issue that radar range equation is inefficient in the near-field region. The precision of the generalized radar equation is verified through the contrast with the numerical simulation. It can also be demonstrated that the proposed equation can be extended from the submillimeter wave band to a much wider band range. Using the near-field radar theory, the proposed equation can be used to estimate the RCS of complicated targets in the THz waveband, which are hard to measure or simulate in the near field; it can also be used to solve the maximum radar range. In short, the near-field radar theory is established, which can be employed in the fine design of the THz radar system and other types of radars applied in the near field.

## Data Availability

The data presented in this study are available on request from the corresponding author.
